# Ribosome preparation from turquoise killifish skeletal muscle for cryo-EM

**DOI:** 10.1016/j.xpro.2021.101087

**Published:** 2022-01-12

**Authors:** Seongsin Lee, Yumi Kim

**Affiliations:** 1 Center for Plant Aging Research, Institute for Basic Science, Daegu 42988, Republic of Korea

**Keywords:** Cell separation/fractionation, Microscopy, Model Organisms, Molecular Biology, Cryo-EM

## Abstract

Ribosomes are composed of a mix of ribosomal RNAs and proteins; this composition varies depending on time, condition, and organism. Here, we present an optimized protocol for preparation of intact ribosomes from the skeletal muscle of the turquoise killifish. We also detail the steps for ribosome quantification and cryo-EM grid preparation. This protocol can enable the identification of heterogeneous ribosome structures that vary by fish age or in response to specific conditions.

## Highlights

• Ribosome enrichment from turquoise killifish skeletal muscle for cryo-EM
• Steps to check quality and quantity of enriched ribosomes
• Preparation of cryo-EM grid with enriched ribosome
• Enables comparison of ribosome structures in varying conditions

## Graphical abstract

**Figure:**
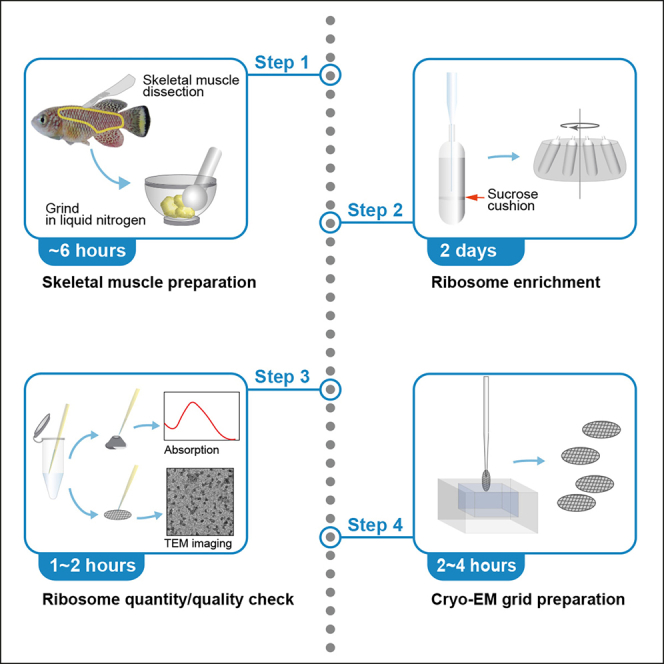

## Before you begin

Skeletal muscle constitutes over 40% of body weight and is critical for supporting body weight and movement and is also the primary site for energy storage. The quality and quantity of muscle changes under diverse conditions such as aging, disease and/or amount of exercise. Ribosome is a major machinery for protein synthesis and highly associated with skeletal muscle quantity. Understanding the *in vivo* functions of ribosomes in skeletal muscle becomes important, such as under the conditions mentioned above. We used the turquoise killifish, which is an emerging model in aging research due to its short lifespan and the high conservation of age-related symptoms and diseases, as well the ease with which it can be exposed to chemical treatments (Kim et al., 2016). Thus, it is important to develop a ribosome preparation protocol for the turquoise killifish to obtain insights into ribosome structure and its functional relevance to aging and disease. The protocol below contains a detailed and optimized procedure preparing intact ribosomes from turquoise killifish skeletal muscle.

## Key resources table

**Table:** 

REAGENT or RESOURCE	SOURCE	IDENTIFIER
**Experimental models: Organisms/strains**		

Nothobranchius furzeri	GRZ-AD	In house strain

**Chemicals, peptides, and recombinant proteins**		

Tricaine methanesulfonate (MS-222)	Sigma-Aldrich	Cat#: E10521-50G
1 M Tris-HCl, pH 7.5	Biosesang	Cat#: TR2016-050-75
Sucrose	Duchefa Biochemie	Cat#: 57-50-1
KCl	Sigma-Aldrich	Cat#: 9541-500G
MgCl_2_	Sigma-Aldrich	Cat#: 208337-100G
Cycloheximide	Merck	Cat#: 239763-1GM
Complete (Protease inhibitor cocktail)	Roche	Cat#: 11836145001
Dithiothreitol (DTT)	Sigma-Aldrich	Cat#: 43815-5G
RNase inhibitor	Invitrogen	Cat#: AM2696
Nuclease-free water	Qiagen	Cat#: 129114
Uranyl acetate solution (1%)	Thomas Scientific	Cat#: 22400-1
Graphene oxide (2 mg/mL)	Sigma-Aldrich	Cat#: 763705

**Software and algorithms**		

NanoDrop 2000/2000c	Thermo Fisher Scientific	Ver1.4.2
ImageJ [DM3 reader]	National Institutes of Health	Ver1.5i

**Others**		

High-speed centrifuge	LaboGene	2236R
GRF-L-c50-6 rotor	LaboGene	GRF-L-c50-6
Centrifuge	LaboGene	1730R
GRF-m2.0-24Lid rotor	LaboGene	GRF-m2.0-24Lid
Optima™ MAX-XP ultracentrifuge	Beckman Coulter	393315
MLA-55 rotor	Beckman Coulter	MLA-55
Cordless tube topper 7700	Beckman Coulter	Cat#: 358312
Seal former, domed top, for tube topper	Beckman Coulter	Cat#: 348120
Ultra-Clear centrifuge tubes	Beckman Coulter	Cat#: 344322
Polypropylene centrifuge tubes	Beckman Coulter	Cat#: 361623
50 mL conical Tubes	Falcon	Cat#: 352070
1.5 mL micro tubes	Sarstedt	Cat#: 72.690.001
10 mL syringes	Shinchang Medical	n/a
0.2 μm syringe filters	Sartorius	Cat#: 16534-k
Disposable glass Pasteur pipettes, 230 mm	Volac	Cat#: D812
Rubber bulbs	SciLab	Cat#: Bul6012
Miracloth	Merck	Cat#: 475855-1R
NanoDrop 2000c	Thermo Fisher Scientific	2000c
Quantifoil R 2/2, UT, 200 mesh, copper	Electron Microscopy Sciences	Cat#: Q250CR2-2nm
Vitrobot™	Thermo Fisher Scientific	n/a

## Materials and equipment

Preparation of the turquoise killifish sacrifice buffer

• Dissolve 1.5 g of tricaine methanosulfonate (MS-222) in 1 L of tank water (pH 7.0, 700 μS).
• Adjust pH to 7.0 with sodium hydrogen carbonate.
• Store the buffer at 4°C and use it within 3 months.
• Warm the solution to 28°C before placing fish in the solution.

Prepare sterilized ribosome isolation buffers by filtering them through 0.2 μm filters.

**Table:** Prepare buffers as follows

Reagent	Homogenization buffer		Sucrose cushion buffer		Washing buffer		Resuspension buffer	
	Final concentration	Amount	Final concentration	Amount	Final concentration	Amount	Final concentration	Amount
KCl (1M)	100 mM	3 mL	100 mM	400 μL	100 mM	500 μL	100 mM	100 μL
MgCl_2_ (1M)	15 mM	450 μL	15 mM	60 μL	15 mM	75 μL	15 mM	15 μL
Tris-HCl pH7.5 (1M)	20 mM	600 μL	20 mM	80 μL	20 mM	100 μL	20 mM	20 μL
Sucrose (2M)	250 mM	3.75 mL	1 M	2 mL	–	0	–	0
Cycloheximide (100×)	1×	300 μL	1×	40 μL	–	0	1×	10 μL
Protease inhibitor cocktail (100×)	1×	300 μL	1×	40 μL	–	0	1×	10 μL
DTT (1M)	1mM	30 μL	–	0	–	0	–	0
Rnase inhibitor (100×)	–	0	–	0	–	0	1×	10 μL
ddH_2_O	n/a	21.57 mL	n/a	1.38 mL	n/a	4.325 mL	n/a	835 μL
Total	n/a	30 mL	n/a	4 mL	n/a	5 mL	n/a	1 mL

***Note:*** Cycloheximide, PI cocktail, DTT and RNase inhibitor should be added just before use.

***Note:*** Buffer compositions were optimized for turquoise killifish muscle by adapting from a previous publication (Forsberg et al., 2017).

## Step-by-step method details

### Preparation of skeletal muscle from the turquoise killifish

**Timing: 2–6 h**

**Figure 1:**
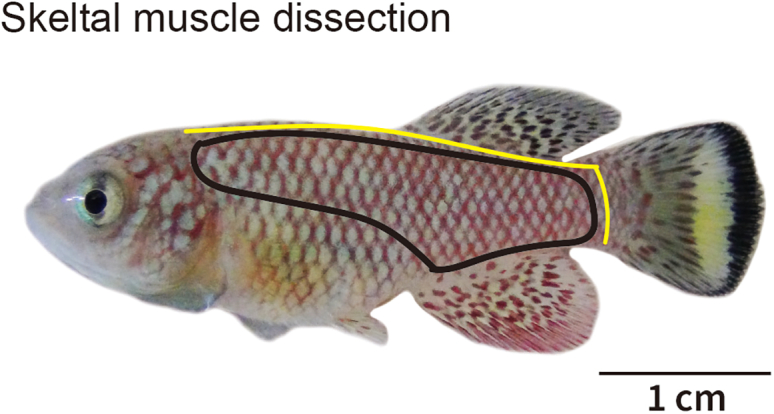
Regional indications for skeletal muscle collection from the turquoise killifish A representative image of male fish at 5 weeks after hatching with a 1 cm scale bar. The yellow line indicates shallow cutting sites for skin removal. The area surrounded by the black line is the region of muscle collection.
3. Collect the skeletal muscle area, indicated as a black line in Figure 1, while avoiding contamination with other tissues.
4. Freeze in liquid nitrogen and store tissue at −80°C before use.
5. Grind the frozen muscle finely (particle size < 100 μm) with a mortar and pestle in liquid nitrogen. [Troubleshooting 2]

***Note:*** Due to the hardness of frozen muscle, we have also tried to grind tissue with a bead beater using different sizes of tungsten beads ranging in diameter from 3 mm to 1 cm. However, the desired fineness was not achieved.

1. Transfer fish (5–6 weeks after hatching, after sexual maturation) to 1.5 g/L tricaine methanosulfonate solution until gill movement stops completely.
2. Remove skin after cutting yellow lines, as shown in Figure 1.

**CRITICAL:** It is hard to measure particle size while grinding, but particle fineness to be optimal when the muscle powder sticks to the mortar wall tightly even in the presence of liquid nitrogen, or you might grind 600 mg of skeletal muscle for 15 min.

6. Add 200 mg aliquots of tissue powder to e-tubes.
7. Store the tubes at −80°C.

### Ribosome purification

**Timing: 2 days**—**Ribosome purification should be performed on ice.**

8. Homogenize 600 mg of finely ground skeletal muscle in 30 mL of homogenization buffer. [Troubleshooting 1]

***Note:*** To purify enough ribosomes for a single Cryo-electron microscopy (EM) experiment, we generally use 600 mg of tissue.

**Figure 2:**
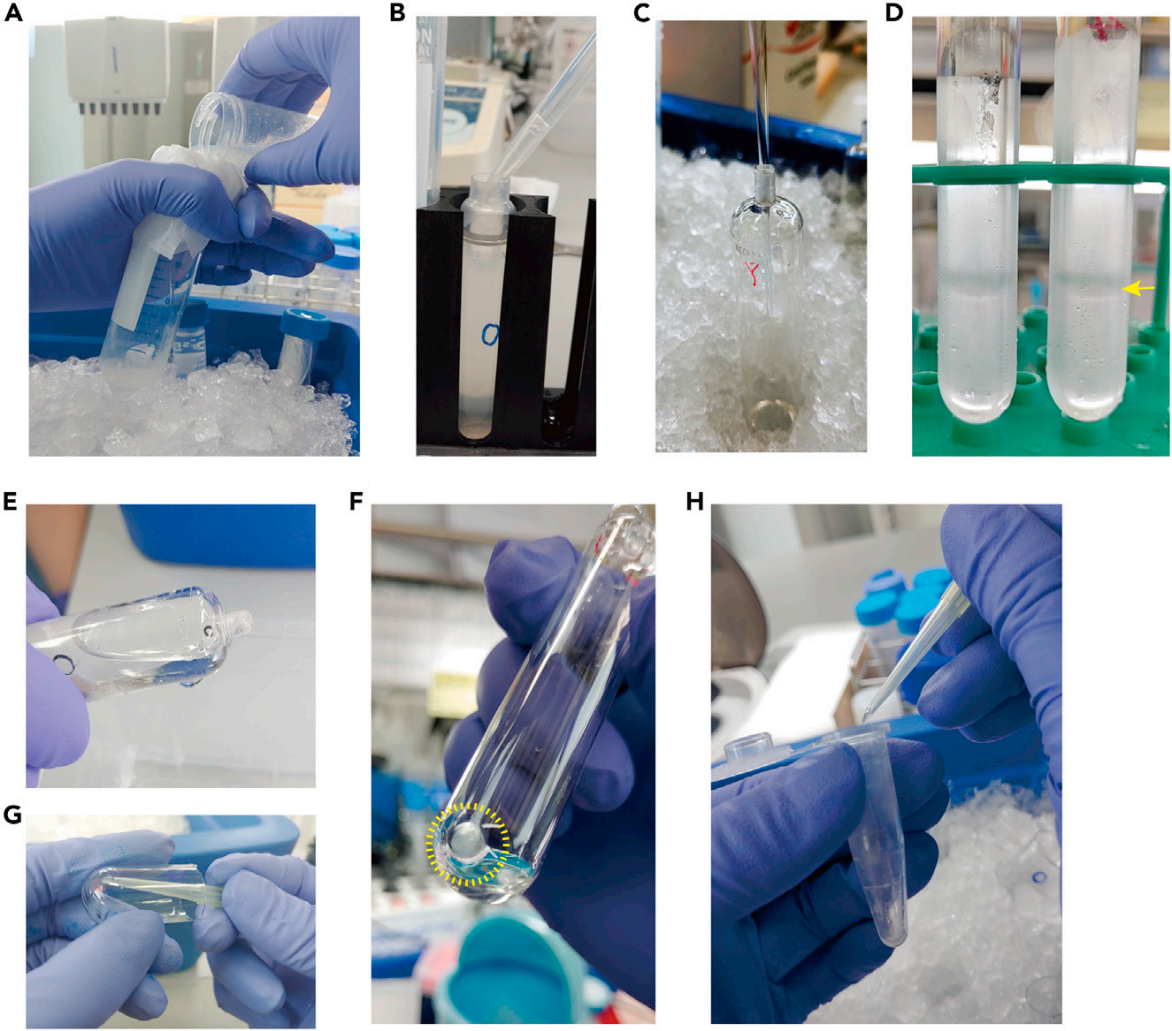
Procedure of ribosome enrichment (A) Filtering of homogenized skeletal muscle. (B) Removal of cell/tissue debris. Homogenized skeletal muscle transferred to a polypropylene centrifuge tube. (C) Preparation of sucrose cushion. Sucrose cushion buffer is filled from the bottom of an ultra-clear centrifuge tube avoiding bubble formation by using a long-tip Pasteur pipette. (D) Loading of protein extract onto the sucrose cushion. The yellow arrow indicates the intact sucrose/protein extract layer. (E) Discarding of the supernatant after centrifugation. (F) Ribosome pellet (yellow-dashed circle) on the bottom of the tube. (G) Collection of ribosome pellet after three washes. (H) Transfer of the ribosome pellet to a new tube.

***Note:*** Collect homogenized solution by squeezing miracloth, thereby minimizing sample loss.

9. Filter homogenized tissue through two layers of miracloth (Figure 2A).

10. Centrifuge at 14,000×*g* for 5 min at 4°C to remove debris.
11. Transfer supernatant into polypropylene centrifuge tubes.
12. Centrifuge at 30,000×*g* for 20 min at 4°C (Figure 2B).
13. Repeat steps 11 and 12.
14. Collect clear supernatant in a 50 mL tube.
15. Prepare sucrose cushion tubes by adding 4 mL of sucrose cushion buffer to ultra-clear centrifuge tubes (Figure 2C).

***Note:*** Because the ultra-clear centrifuge tubes have a narrow neck, it is much easier to use a Pasteur pipet as a funnel for filling sucrose from the bottom.

16. Load cleared solution from step 14 onto sucrose cushion. Make sure not to disrupt the sucrose cushion layer (Figure 2D).
17. Heat-seal the top of the tube after balancing with a seal former and cordless tube topper.
18. Centrifuge at 150,000×*g* for 12 h at 4°C.
19. Remove the supernatant very carefully so as not to lose the ribosome pellet (Figure 2E).
20. Very carefully wash the pellets three times with 1 mL of washing buffer.

***Note:*** Make the washing buffer flow the surface of the pellet couple of times per wash. Try not to touch pellet while washing.

**CRITICAL:** It is easy to lose a ribosome pellet in this step, so make sure that the ribosome pellet stays on the tube wall every wash. **[**Troubleshooting 2**]**

21. Check to see if a transparent ribosome pellet is visible at the bottom of the tube (Figure 2F).
22. Collect ribosome pellets with a tip and resuspend them in 50 μL of resuspension buffer (Figures 2G and 2H).

***Note:*** The pipette tip should be cut diagonally for easier collection of the ribosome pellet. The ribosome pellet is sticky, making it difficult to collect from the tube. Transferring a ribosome pellet can be facilitated by touching the e-tube wall. The remaining ribosome pellets should be collected by adding resuspension buffer directly to the centrifuge tube.

23. Gently dissolve ribosome pellets by pipetting, while avoiding bubble formation. [Troubleshooting 3]
24. Centrifuge at 20,000×*g* for 10 min at 4°C.
25. Transfer the clear supernatant to a new tube.

### Ribosome purity check

**Timing: 1–2 h**

This step determines the quality and quantity of the isolated ribosomes prior to making a cryo-EM grid.

**Figure 3:**
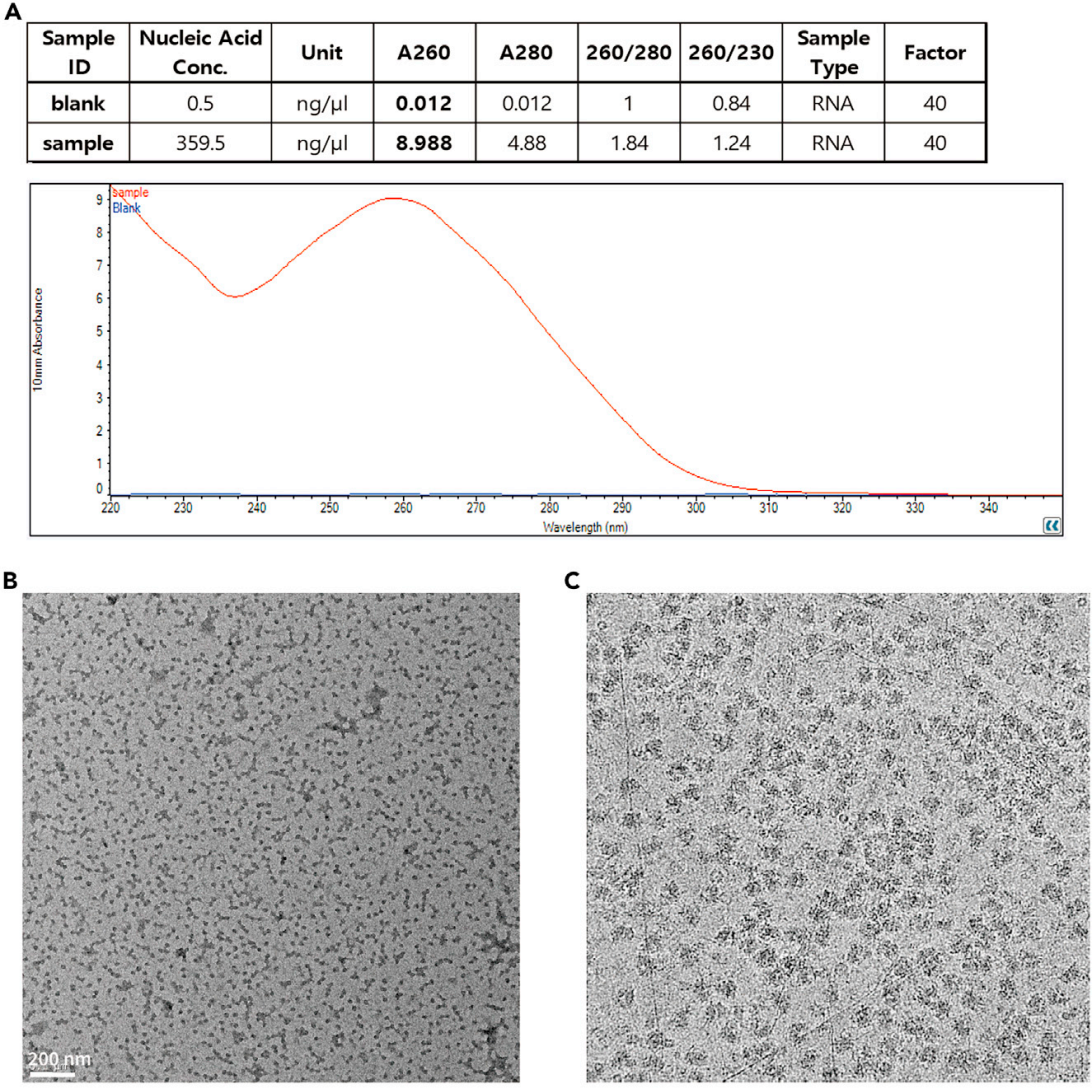
Enriched ribosomes (A) Determination of ribosome quantity and purity by measuring RNA concentration. A260 should be over 8 and wave-length dependent absorbance should be checked shown as in the Figure. (B) Contamination check by TEM. (C) Reference image of ribosomes by Cryo-EM.

***Note:*** OD 8–12 at 260 nm is a good quantity range. If OD is higher than 12, adjust ribosome into OD 8–12 with resuspension buffer.

26. Measure optical density (OD) at 260 nm (Figure 3A).

27. Assess ribosome purity by transmission electron microscopy (TEM) (tecnai G2 F20, ThermoFisher) (Figure 3B).
  a. Glow discharge a grid (Quantifoil R2/2) with argon gas for 30 sec.
    ***Note:*** We used the Gatan Plasma System (US100, Gatan) using the instructions on the screen of the equipment.

**Table:** 

Title	Ar PLASMA	O2 Gas Flow	0.0 sccm
Visible	Yes	H2 Gas Flow	0.0 sccm
Cleaning Time	0:25	Ar Gas Flow	30.0 sccm
Vacuum Target	70 mTorr	Gas Flow Timeout	20 seconds
Vacuum Range	0 m Torr	Forward RF Target	50 W
Pumping Switch Point	20 Torr	Forward RF Range	5 W
Turbo Pump Speed	750 Hz	Maximum Reflected RF	5 W
Pumping Timeout	120 seconds	RF Tuning Timeout	4 seconds
Repeat	No	RF Tuning Attempts	3
  b. Load 4 μL of isolated ribosome onto the grid and incubate for 30 sec.
  c. Wash the grid twice by placing the grid briefly onto a drop of distilled water.
  d. Remove excess water from the grid by touching the grid edge with filter paper.
  e. Incubate the grid in uranyl acetate for 30 sec.
  f. Wash the grid twice by placing the grid briefly onto a drop of distilled water.
  g. Remove excess water from the grid by touching the grid edge with filter paper.
  h. Dry the grid for 5 min at 25°C.
    ***Note:*** Ribosomes usually stained darker than background in our trials. It is critical to check ribosome particle distribution, aggregation and contamination.

### Preparation of cryo-EM grid

**Timing: 2–4 h**

This step describes preparing grids for cryo-EM. This step can be adjusted according to sample condition.

28. Glow discharge both sides of a new grid with argon gas for 30 s which is the same condition above (step 27a).
29. Coat the grid with graphene oxide. [Troubleshooting 4]
  a. Load 3 μL of graphene oxide solution onto the carbon side and incubate for 2 min
  b. Remove excess graphene oxide from the grid by touching the grid edge with filter paper and wash three times by placing the grid on distilled water drop.
  c. Remove excess water on the grid touching grid edge to filter paper.
  d. Dry for 5 min and use within a month without additional glow-discharge.

***Note:*** This step is performed according to a described protocol (Bokori-Brown et al., 2016).

30. Cryo-EM grid preparation
  a. Set-up Vitrobot for Cryo-EM grid preparation.
    ***Note:*** We used VitrobotTM (FEI, Thermo Fisher Scientific) using the process parameters on the screen of the equipment.

**Table:** 

Blot time (s)	3.0	Blot force	10
Wait time (s)	30.0	Blot total	1
Drain time (s)	0.0	Skip application
  b. Load 3 μL of sample onto the graphene oxide coated grid.
  c. Wait 30 s and blot the ribosomes for 3 s with 10 of blotting force.
    ***Note:*** When you are trying to optimize sample blotting conditions for a new sample, it is recommended that 6–12 grids be prepared per sample and that different sample concentrations and waiting and blotting times to be tested.

31. Store grids in liquid nitrogen before imaging and data collection.
32. Collect data (Figure 3C).

## Expected outcomes

**Figure 4:**
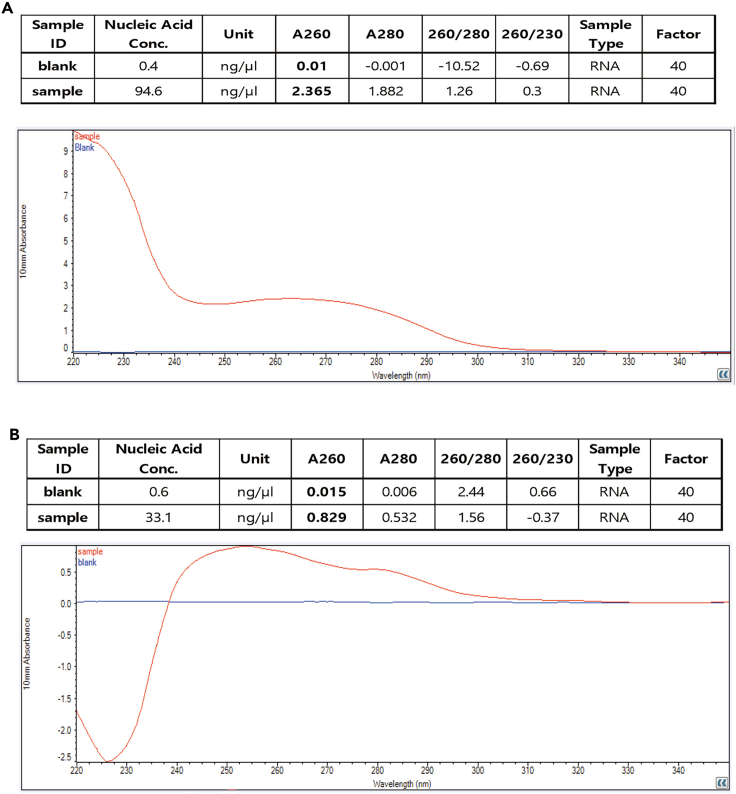
Failure of ribosome enrichment (A) Representative absorption plot resulting from a lack of sufficient starting muscle tissue. (B) Representative absorption plot resulting from a loss of ribosome pellet.

**Figure 5:**
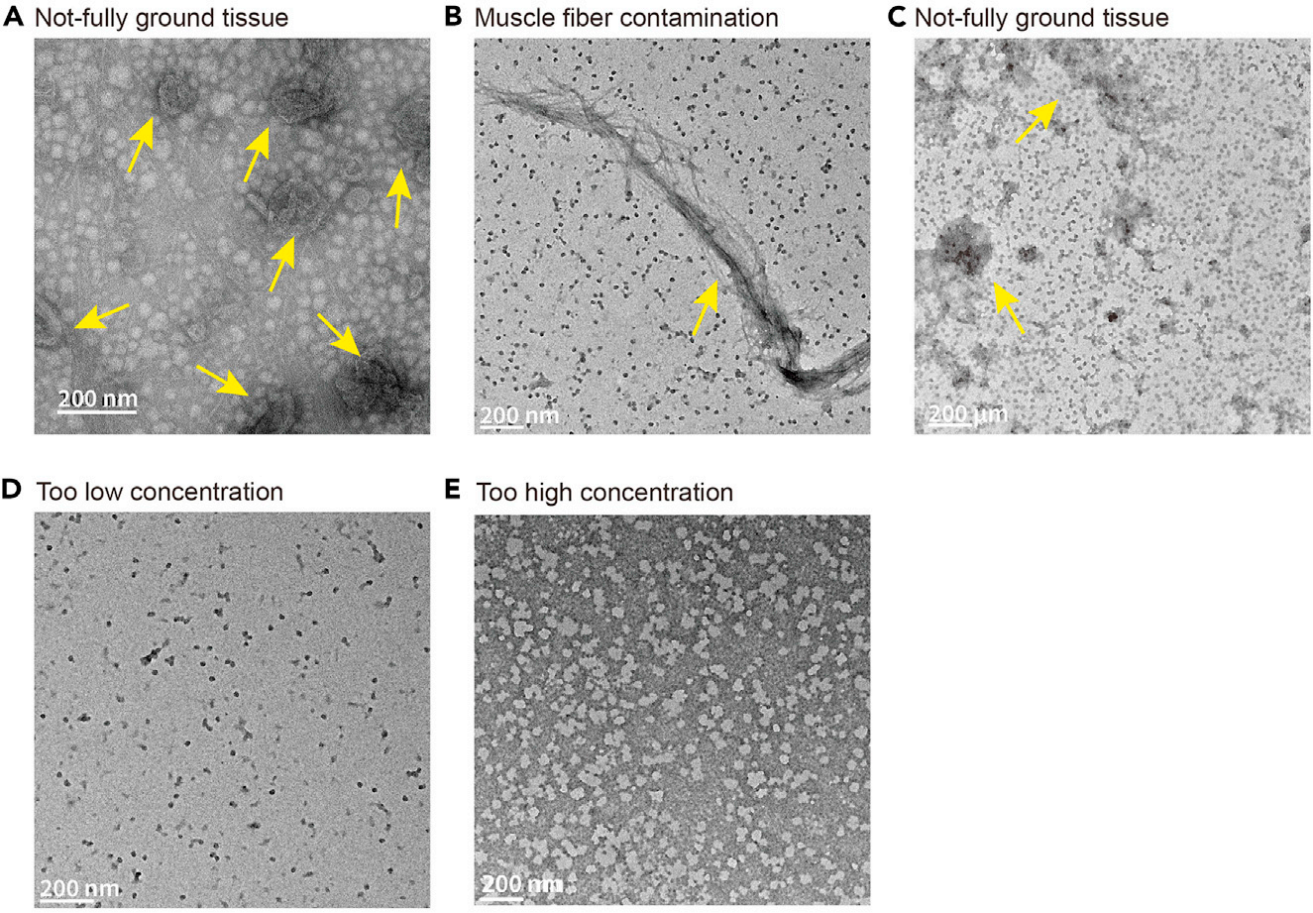
Examples of ribosome preparation failure at each critical step (A) TEM image of ribosomes from incompletely ground muscle tissue (yellow arrows). Related to Step 3 in the preparation of skeletal muscle from the turquoise killifish. (B) TEM image of ribosomes contaminated by muscle fibers (red arrow). Related to steps 9–14. (C) TEM image of aggregated ribosomes (green arrows). Related to step 23. (D) Ribosome particles at too low concentration. (E) Ribosome particles at too high concentration.

This protocol describes the overall procedure from skeletal muscle sample to cryo-EM grid preparation. The quantity and quality of isolated ribosomes can be monitored in steps 26 and 27 (Figures 3, 4, and 5). These steps are critical to proceed to the next steps for Cryo-EM data collection. If the ribosome quantity and quality do not meet the suggested criteria, further steps are not warranted. Furthermore, graphene oxide coating also greatly affects ribosome loading onto the grid. Graphene oxide coatings are highly variable, and depending on the situation, which can cause different degrees of grid quality. Thus, it is necessary to determine whether one or two grids are coated with graphene oxide prior to sample loading. In addition, the best grids and holes should be screened with a microscope after sample loading. Ribosomes on grids can be imaged and data can be collected as shown in Figure 3C. This protocol helps to reduce errors that can occur during ribosome sample preparation from skeletal muscle. Tissue properties differ, making it necessary to adjust experimental conditions for different tissues.

## Limitations

Ribosome preparation from animal tissue is largely dependent on the amount and properties of the tissue of interest. The turquoise killifish is a small teleost fish ranging from 2.5–6 cm in length. Collecting skeletal muscle tissue from young fish (5–6 weeks after hatching, after sexual maturation) is difficult due to its softness and small quantity. By contrast, harvesting skeletal muscle from old fish (16–18 weeks after hatching, around the median lifespan) is easier. However, ribosome yield from the muscle of old fish is generally lower than its yield from the muscle of young fish for unknown reasons. These variables affect sample size for the experiments. EM may be a limiting step to check ribosome quality and grid preparation. Finding the closest EM facility is always best when preparing grids. Ribosomes obtained under certain conditions, such as those from old fish, are unstable based on our experiences; thus, grids should be prepared immediately after ribosome isolation.

## Troubleshooting

### Problem 1

Low yield of ribosomes (Figure 4).

### Potential solution

Ribosome yield can be dependent on sample quantity (Figure 4A). To increase yield, use larger tissue samples, while increasing the starting sample amount and volumes of the homogenization buffers accordingly. Importantly, do not mix ribosomes isolated on different days. Ribosome pellets can also be lost when washing and transferring them to new tubes (Figure 4B). In this case, the positions of ribosome pellets should be marked on the centrifuge tubes, to ensure the transfer of sticky ribosome pellets.

### Problem 2

Contaminated ribosomes.

### Potential solution

Not fully-ground muscle tissue and myosin are major sources of contamination (Figures 5A and 5B). Muscle tissue should be ground as finely as possible. Muscle fiber contamination often occurs when the volume of homogenization buffer is insufficient. An optimal amount is 600 mg tissue per 30 mL of homogenization buffer. Increasing the volume of the homogenization buffer reduces muscle fiber contamination. The volume of this buffer, however, can be adjusted as needed.

### Problem 3

Aggregated ribosomes.

### Potential solution

Ribosomes often aggregate when the ribosome pellet is not fully suspended (Figure 5C). Sufficient time is needed for careful resuspension of ribosome pellets. Fully suspended ribosomes at the optimal concentration should resemble those shown in Figure 3B. Fully suspended ribosomes at low and high concentrations should be resemble those shown like in Figures 5D and 5E, respectively.

### Problem 4

Cracked grids and wrinkled carbon film.

### Potential solution

**Figure 6:**
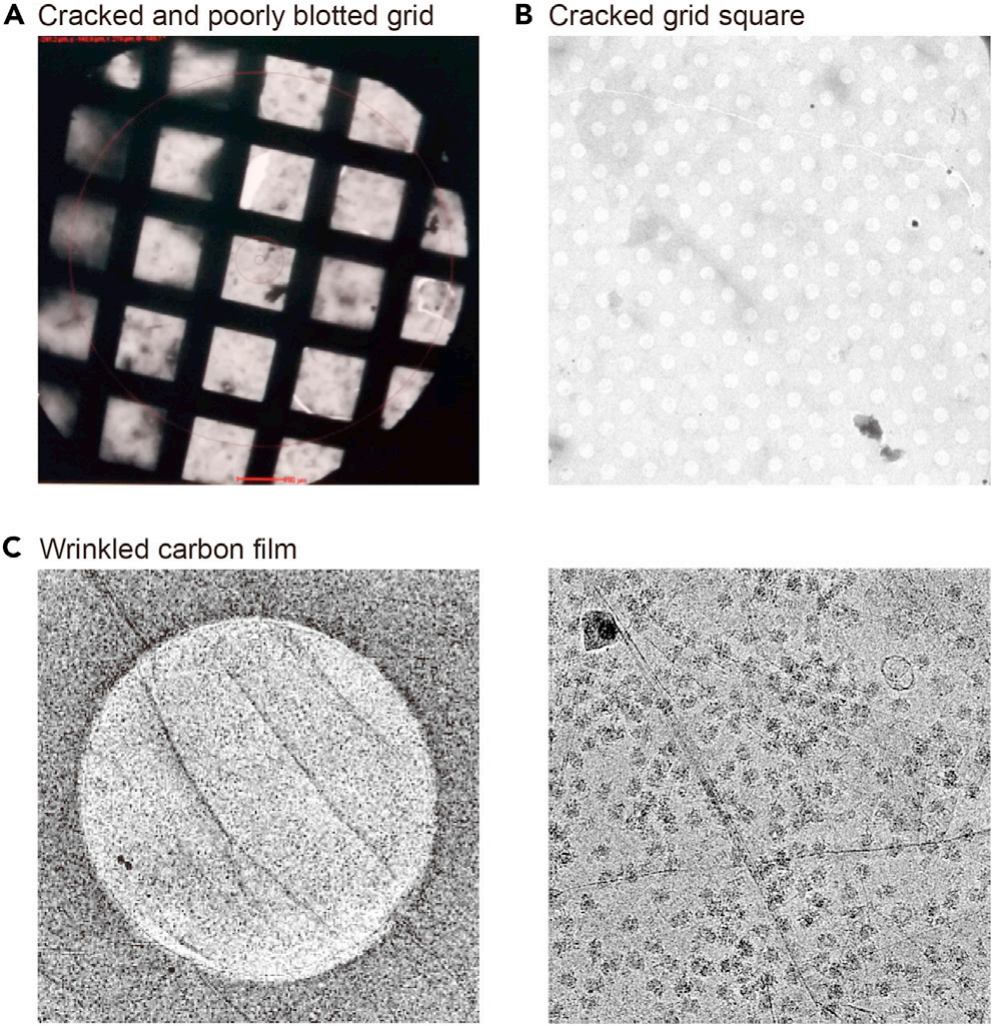
Examples of poorly prepared EM grids (A) A cracked and un-evenly blotted grid. (B) Grid square containing a crack and dust. (C) Grid foil hole with wrinkled carbon film and micrograph.

Cracked grids are often observed during cryo-EM data collection (Figures 6A and 6B). This occurs during blotting ribosome samples to grid. Usually, cracked girds can be discarded at the final screening step. However, if this occurs to all grids with no useful squares, blotting force should be reduced. Wrinkled carbon film is frequently observed when the grid is coated by graphene oxide (Figure 6C). Alternatively, carbon coating can be performed using plasma deposition. Either method is appropriate if it results in a single layer of carbon on the grid.

## Resource availability

### Lead contact

Further information and requests for resources and reagents should be directed to and will be fulfilled by the lead contact, Yumi Kim (yumikim@ibs.re.kr).

### Materials availability

This protocol did not generate new materials.

## Data Availability

The data from this study are available from the corresponding author upon reasonable request. This study did not generate any new codes.
